# *An MSH6* germline pathogenic variant p.Gly162Ter associated with Lynch syndrome

**DOI:** 10.1038/s41439-022-00216-7

**Published:** 2022-10-26

**Authors:** Olga A. Vostrukhina, Elena D. Mirlina, Darya N. Khmelkova, Galina M. Butrovich, Alexandra D. Shakhmatova, Yury V. Kil, Yliya L. Polyatskin, Anna S. Artemyeva, Alexey V. Gulyaev, Valery N. Verbenko

**Affiliations:** 1 Petersburg Nuclear Physics Institute named by B.P. Konstantinov of National Research Centre “Kurchatov Institute”, Gatchina, 188300 Russia; 2 Centre of Genetics and Reproductive Medicine “Genetico”, Moscow, 119333 Russia; 3 N.N. Petrov National Medical Research Centre of Oncology, St. Petersburg, 197758 Russia

**Keywords:** Cancer genetics, Oncogenes

## Abstract

We identified a three-generation Russian family with Lynch syndrome with a novel germline variant of the *MSH6* gene. An 84-year-old female was diagnosed with endometrial adenocarcinoma at the age of 49 years. Her son was diagnosed with colorectal tubular adenoma at the age of 32 years. A germline nonsense variant (c.484 G > T:p.Gly162Ter) in exon 3 of the *MSH6* gene was revealed by whole-exome sequencing. Sanger sequencing confirmed the cosegregation of the *MSH6* nonsense variant in family members.

Lynch syndrome (LS; OMIM#120435) is one of the manifestations of hereditary nonpolyposis colorectal cancer syndrome (HNPCC) and is associated with a genetic predisposition to different cancer types, including colon cancer, endometrial cancer, ovarian cancer, stomach cancer, and cancer of the small bowel, pancreas, and hepatobiliary tract^1^. In addition, data indicate a mildly increased risk of breast cancer^2^. HNPCC disorders show a proclivity to early onset and an excess of multiple primary tumors.

Dysfunction of DNA mismatch repair (MMR) leads to an increased frequency of biosynthetic errors generated during DNA replication and plays a critical role in the accumulation of mutations in cancer-related genes. LS results from a loss-of-function germline mutation in one of four different genes (*MLH1*, *MSH2*, *MSH6*, and *PMS2*) encoding mismatch repair proteins^3^. Mutations in the MMR genes *MLH1*, *MSH2*, *MSH6*, and *PMS2* account for 40, 34, 18 and 8% of LS patients, respectively^4^. *EPCAM* deletion-associated hypermethylation of the *MSH2* promoter varies in frequency between populations and may account for 10–40% of families with absent MSH2 protein in tumors^5^. A very small number of variants are in the more recently identified *MSH3*^6^. The mismatch repair deficiency was shown to be frequently accompanied by microsatellite instability in tumors^7^. Two heterodimeric complexes, MSH2/MSH6 and MSH2/MSH3 (MutSα and MutSβ, respectively)^8,9^, are involved in mismatch recognition and initiation of repair^10^. MLH1 forms a complex with PMS2 and functions as an endonuclease.

In this study, we describe a new pathogenic germline mutation in the *MSH6* gene revealed by whole-exome sequencing (WES) in the genome of a proband of a three-generation family from northwestern Russia diagnosed with LS. An 84-year-old female who was diagnosed with endometrial adenocarcinoma at the age of 49 years was the proband (I) (Fig. 1). Between the ages of 49 and 79 years, the female patient developed an additional six primary malignant lesions: four were located in the intestine, and two were located in the breasts. She was successfully treated with surgery. Immunohistochemistry and mutational analysis showed that the lesions had different microsatellite stability, and the APC, KRAS, TGFBR2, TP53, PIC3CA, ARID1A, and e-cadherin status was not uniform among the lesions. Now, the affected family members include 2 individuals. Three members of the family (I, II, and III-1) provided peripheral blood samples and clinical information. The medical history was further investigated for disease occurrence. II is a 48-year-old man diagnosed with colorectal tubular adenoma of the sigmoid colon who underwent surgery at the age of 32 years. III-1 is a 21-year-old healthy female. The father of the proband died from colon cancer at the age of 52 years, and her mother died from breast cancer at the same age.

**Fig. 1:**
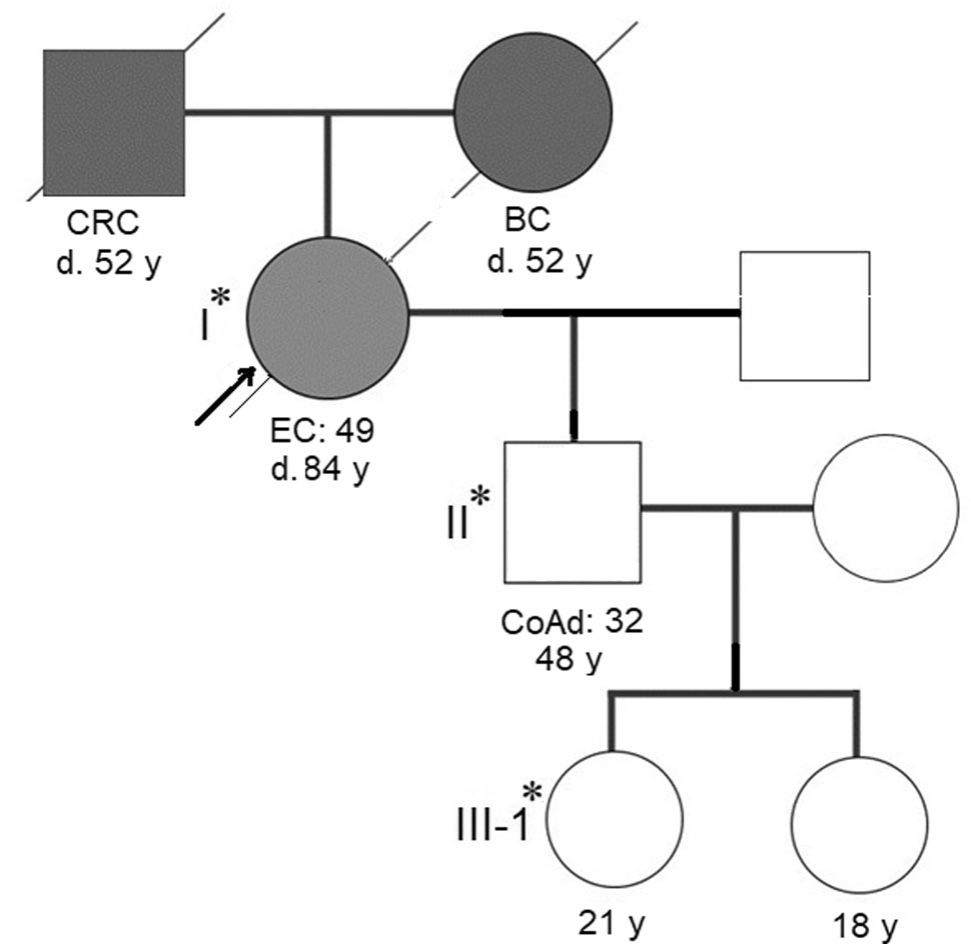
Pedigree of a three-generation Russian family with Lynch syndrome. Asterisks depict the family members who provided blood samples. CRC colon cancer, BC breast cancer, EC endometrium adenocarcinoma, CoAd colorectal tubular adenoma of the sigmoid colon.

As we can see, the family history fulfilled the Amsterdam II criteria for LS: three family members were affected with LS-associated cancers, all of them were first-degree relatives, two generations were affected, and the proband was diagnosed before age 50^11^.

WES has enabled the identification of the gene responsible for LS in this family. WES of the blood sample from affected family member I was used by Genetico (Moscow, Russia). The Illumina NovaSeq 6000 platform (Illumina Inc., San Diego, CA, USA) was utilized for WES with a 100-bp paired-end run protocol. The WES results revealed several thousand synonymous and missense SNVs, deletions, insertions, duplications, and other variants in the proband’s genome. The variants of genes associated with the respective cancer predisposition syndrome, including Lynch syndrome, adenomatous polyposis and hamartomatous polyposis syndromes, hereditary breast-ovarian cancer, hereditary diffuse gastric cancer, Li-Fraumeni syndrome and other cancer-associated syndromes^2,12–14^, were searched for pathogenic mutations. Only variants with a minor allele frequency (MAF) < 0.01 in the Ensembl Project, ExAC browser, and gnomAD databases were included.

As a result, a stop-gain variant at chr2:28023059 G > T (GRCH37/hg19) in the mismatch repair gene *MSH6* (NM_000179.3:c.484 G > T) was identified. This mutation is expected to result in termination of the amino acid chain after lysine-161 (p.Gly162Ter) in the N-terminal domain of the MSH6 protein. To our knowledge, the nonsense variant c.484 G > T in exon 3 of the *MSH6* gene is absent from all population databases. It was not found in the Ensembl Project, ClinVar, or gnomAD, and it was not reported for LS previously. Only missense variant c.484 G > A:p.Gly162Arg with uncertain significance is present in ClinVar and gnomAD. As mismatch repair genes, including *MSH6*, are considered to be involved in tumorigenesis, the nonsense variant of *MSH6*, c.484 G > T, was subjected to further investigation.

This mutation was verified by Sanger sequencing (Fig. 2A). The segregation analysis of the mutation in the *MSH6* gene among other family members was performed by PCR amplification and Sanger sequencing of targeted DNA fragments from two individuals, including one patient (II) and one unaffected family member (III-1). PCR products were sequenced by Genetico (Moscow, Russia). In addition to the proband, both family members analyzed (II and III-1) carried the nonsense variant *MSH6*: c.484 G > T (Fig. 2B and Fig. 2C, respectively).

**Fig. 2:**
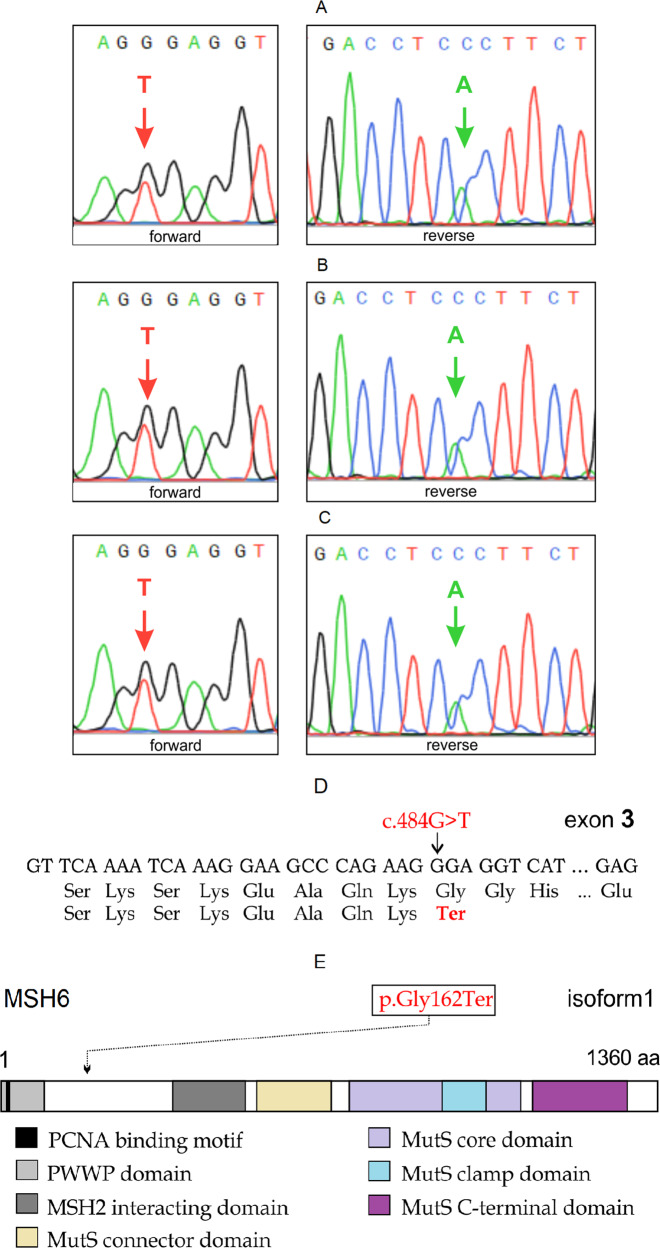
Sanger sequencing results. A portion of the sequence chromatogram from MSH6 exon 3 demonstrates a heterozygous variant, c.484 G > T, in proband I (**A**), affected family member II (**B**) and unaffected family member III-1 (**C**). The reading in both directions is presented. The position of the nonsense mutation c.484 G > T [p.Gly162Ter] in the frame of exon 3 (**D**) and a schematic representation of the MSH6 protein (**E**) are shown.

The stop codon (Fig. 2D) eliminates almost all functional motifs: MSH2 interacting domain, MutS connector, core, clamp, and C-terminal domains (Fig. 2E). To date, it is known that c.467 C > G:p.S156Ter stop-gain mutation is associated with colon tumors, bladder tumors, and adenomatous polyps^15^. The S144I substitution causes inherited somatic deficiency in MMR, resulting in increased development of HNPCC^16^.

The ACMG/AMP classification^17,18^ for assessing the pathogenicity of different variants allowed us to estimate the nonsense variant (c.484 G > T) in exon 3 of *MSH6*. The null variant occurs in a gene where loss of function is a known mechanism of disease (PVS1)^15^; is absent in population databases (PM2) (allele frequency = 0 in Ensembl Project, ClinVar, and gnomAD); and cosegregates in affected members of the family (PP1) (this work). Moreover, Cancer Genome Interpreter (www.cancergenomeinterpreter.org) identified the *MSH6* c.484 G > T variant as an oncogenic mutation. In summary, the nonsense variant (c.484 G > T) of *MSH6* fulfilled the criteria of ACMG for a “pathogenic variant”, as one very strong (PVS1), one moderate (PM2), and one supportive (PP1) criteria of pathogenicity have been revealed for this germline variant.

Previous studies also reported nonsense variants of the *MSH6* gene in Lynch syndrome families that were classified as deleterious: *MSH6* c.1030 C > T:p.Gln344Ter encodes a protein with loss of 1017 amino acid residues^19^, pSer156Ter is associated with endometrial cancer; Gln177Ter is associated with LS, Ser200Ter is reported in LS or CRC, and Glu207Ter is associated with NPCRC or LSL in the HGMD; and Ser200Ter is reported in LS or CRC in gnomAD.

In conclusion, we reported a novel nonsense variant (c.484 G > T:p.Gly162Ter) in exon 3 of *MSH6* (NM_000179.3). Clinical data, cosegregation analysis, and in silico prediction convincingly classified the *MSH6* variant (c.484 G > T) as pathogenic and the loss-of-function mutation for LS in the family. Therefore, our results broaden the genotypic range of *MSH6* mutations that cause LS. Visual invasive examinations, such as CT colonography, flexible sigmoidoscopy or colonoscopy every 1–2 years, were recommended to one unaffected family member (III-1) who also carried the *MSH6* variant c.484 G > T.

## HGV Database

The relevant data from this Data Report are hosted at the Human Genome Variation Database at 10.6084/m9.figshare.hgv.3243.
